# Prevalence of first adolescent pregnancy and its associated factors in sub-Saharan Africa: A multi-country analysis

**DOI:** 10.1371/journal.pone.0246308

**Published:** 2021-02-04

**Authors:** Bright Opoku Ahinkorah, Melissa Kang, Lin Perry, Fiona Brooks, Andrew Hayen

**Affiliations:** 1 School of Public Health, Faculty of Health, University of Technology Sydney, Ultimo, New South Wales, Australia; 2 Faculty of Health, University of Technology Sydney, Ultimo, New South Wales, Australia; University of Salamanca, SPAIN

## Abstract

**Introduction:**

In low-and middle-income countries, pregnancy-related complications are major causes of death for young women. This study aimed to determine the prevalence of first adolescent pregnancy and its associated factors in sub-Saharan Africa.

**Methods:**

We undertook a secondary analysis of cross-sectional data from Demographic and Health Surveys conducted in 32 sub-Saharan African countries between 2010 and 2018. We calculated the prevalence of first adolescent (aged 15 to 19 years) pregnancy in each country and examined associations between individual and contextual level factors and first adolescent pregnancy.

**Results:**

Among all adolescents, Congo experienced the highest prevalence of first adolescent pregnancy (44.3%) and Rwanda the lowest (7.2%). However, among adolescents who had ever had sex, the prevalence ranged from 36.5% in Rwanda to 75.6% in Chad. The odds of first adolescent pregnancy was higher with increasing age, working, being married/cohabiting, having primary education only, early sexual initiation, knowledge of contraceptives, no unmet need for contraception and poorest wealth quintile. By contrast, adolescents who lived in rural areas and in the West African sub-region had lower odds of first adolescent pregnancy.

**Conclusion:**

The prevalence of adolescent pregnancy in sub-Saharan African countries is high. Understanding the predictors of first adolescent pregnancy can facilitate the development of effective social policies such as family planning and comprehensive sex and relationship education in sub-Saharan Africa and can help ensure healthy lives and promotion of well-being for adolescents and their families and communities.

## Introduction

Pregnancy among adolescent girls (aged 15 to19 years) is often associated with high risks to both the mother and the fetus [1] and can lead to intergenerational cycles of poverty, poor education and unemployment [2]. In low-and middle-income countries, pregnancy-related complications are major causes of death for girls aged 15 to 19 years old [3].

Globally, adolescent birth rates have fallen from 65 births per 1000 women in 1990 to 47 births per 1000 women in 2015 [4]. In 2014, Sedgh, Finer [5] provided a comprehensive overview of the variations in adolescent pregnancy across countries by looking at the trends of adolescent pregnancy, birth and abortion rate and concluded that despite recent declines, adolescent pregnancy rates remain high in many countries. The number of adolescent pregnancies is projected to increase globally by 2030, as the total population of adolescents continues to grow, with the greatest proportional increases in Western and Central, Eastern and Southern Africa [6]. The projected increase in adolescent pregnancies is likely to be more prevalent in sub-Saharan Africa (SSA), which already leads the world in teen pregnancies [7, 8] and child marriage [9].

Efforts have been made to reduce adolescent pregnancy globally, and this is evident in the Sustainable Development Goal 3, Target 3.7 that seeks to ensure universal access to sexual and reproductive health-care services, including family planning, information and education, and the integration of reproductive health into national strategies and programmes by 2030 [10]. This is important in respect to the high rates of maternal mortality [11], abortion [12] and neonatal deaths [13] associated with adolescent pregnancy in SSA. International evidence links the provision of high quality comprehensive sex and relationship education to improved use of contraception as major strategies for addressing adolescent pregnancy [14]. In SSA, many programs and strategies, including comprehensive sex education and family planning services are geared towards reduction in adolescent pregnancy [15–17]. However, their impact to date is unclear, as adolescent pregnancy rates remain high in countries in SSA [18].

The effectiveness of these programs and strategies depends on multiple factors, but empirical evidence is not always available for all the potential predictors of adolescent pregnancy in the sub-Saharan African region. In this sub-region, most studies have focused on single countries only [19–23], with few using nationally-representative data from multiple countries [24, 25]. Others have combined the findings of single country studies and examined the predictors of adolescent pregnancy through systematic reviews and meta-analyses [26–28]. These studies have identified sexual coercion or pressure from male partners, low or incorrect use of contraceptives, lack of parental communication and support, early marriage, religion, early sexual debut, lack of comprehensive sexuality education, residence, marital status, low self-esteem and educational status of adolescents [26–28] as correlates of adolescent pregnancy. However, major issues in these previous analyses include the use of outdated data, from as far back as 2001 [27], and the combination of data which are nationally-representative with those from selected areas within single countries [26–28]. No other publications have combined the findings of studies carried out in all countries in SSA using Demographic and Health Survey (DHS) data. Since adolescent pregnancy is a major phenomenon in SSA, examining its prevalence and predictors in multiple countries can help understand the patterns of prevalence and common predictors across the countries of SSA. We, therefore, sought to fill these gaps by examining the prevalence of first adolescent pregnancy and its associated factors in SSA using nationally representative data from 32 countries collected between 2010 and 2018. Examination of factors associated with first adolescent pregnancy in multiple countries using DHS in this sub-region can help develop common strategies for dealing with adolescent pregnancy across the sub-region. Furthermore, large-scale, nationally representative surveys such as DHS provide opportunities for many countries to have more comprehensive information on adolescent fertility that assimilates some of the contextual, socio-economic and geographic factors [29]. Findings from the study will also enhance the evidence available to inform policy and practice development towards achieving Sustainable Development Goal 3 which seeks to ensure healthy lives and promote well-being for all at all ages [10].

## Methods

### Design and sampling

We conducted a secondary analysis of data from the DHS conducted between January 1 2010 and December 31 2018 in 32 countries in SSA. The DHS is a nationwide survey mostly collected every five-year period across low-and middle-income countries. It uses standard procedures for sampling, questionnaires, data collection, cleaning, coding and analysis, which allows for cross-country comparison [30]. The survey employs a stratified two-stage sampling technique [31]. The first stage involves the development of a sampling frame, consisting of a list of primary sampling units (PSUs) or enumeration areas (EAs), which covers the entire country and is usually developed from the latest available national census. The second stage is the systematic sampling of households listed in each cluster or EA. In this study, we first accessed data on a total of 95,703 female adolescents (15–19 years) from 32 countries in SSA to analyse the prevalence of first adolescent pregnancy among all adolescents in SSA (see Table 1). For subsequent analysis, we excluded adolescents who had never had sex and examined the prevalence and predictors of first adolescent pregnancy among adolescents who had ever had sex. Within this subset, there were complete data available for the included variables of interest for 40,272 female adolescents. We included all who provided an age at first sex, while excluding those who responded that they had never had sex. The rationale was to examine the factors associated with first adolescent pregnancy among those adolescents who are at risk of getting pregnant through sexual initiation.

**Table 1:** Prevalence of adolescent pregnancy in 32 sub-Saharan African countries (DHS, 2010–2018).

Country	Year of survey	All adolescents; n	Adolescents who had ever had sex; n	Any first pregnancy^a^ %	Any first pregnancy^b^ %	Pregnant at the time of the survey^b^ %	Ever given birth^b^ %	Ever had a pregnancy terminated^b^ %
Angola	2015–16	3363	2117	39.4	58.1	13.9	46.6	4.5
Benin	2017–18	3335	1624	21.1	43.9	13.2	31.7	4.7
Burkina Faso	2010	3347	1429	23.5	55.6	15.4	42.3	4.7
Burundi	2016–17	3968	549	7.9	58.7	19.2	42.2	4.1
Cameroon	2011	3579	1753	28.3	51.9	13.5	40.2	8.0
Chad	2014–15	3874	1785	35.9	75.6	8.2	62.1	6.1
Comoros	2012	1291	244	11.3	57.2	15.2	49.8	4.6
Congo	2011–12	2163	1396	44.3	56.4	12.3	42.3	13.9
Congo DR	2013–14	3980	2090	31.2	53.9	15.6	40.3	4.1
Côte d’Ivoire	2011–12	1995	1283	32.2	51.9	13.4	34.4	11.5
Ethiopia	2016	3498	842	13.3	52.5	12.2	40.9	3.7
Gabon	2012	1833	1129	38.0	47.5	11.4	33.7	11.4
Gambia	2013	2461	580	19.6	73.4	18.8	58.0	6.5
Ghana	2014	1756	698	15.1	38.0	8.9	25.9	7.7
Guinea	2018	2561	1102	27.6	65.6	16.9	48.5	6.2
Kenya	2014	2861	974	18.2	49.7	15.4	39.2	2.7
Lesotho	2014	1542	664	19.8	43.2	9.6	32.7	2.4
Liberia	2013	1914	1441	38.9	46.1	11.4	36.0	4.1
Malawi	2015–16	5273	2745	29.3	58.1	15.7	42.5	4.4
Mali	2018	2209	1223	36.3	64.1	16.8	52.1	5.1
Namibia	2013	1857	847	21.0	42.2	12.5	30.2	1.9
Niger	2012	1901	1131	37.1	67.3	20.0	52.3	8.0
Nigeria	2018	8423	1364	19.0	53.3	15.5	41.4	5.0
Rwanda	2014–15	2779	558	7.2	36.5	9.2	27.5	1.8
Senegal	2010–11	3604	930	22.3	68.5	16.4	54.3	7.1
Sierra Leone	2013	4050	2400	28.3	43.8	11.1	33.1	3.5
South Africa	2016	1505	620	16.9	37.1	7.7	28.2	2.0
Tanzania	2015–16	2931	1531	25.1	53.4	15.6	40.2	5.5
Togo	2013–14	1732	784	18.2	37.3	8.2	28.1	4.3
Uganda	2016	4276	1958	26.4	56.6	17.2	42.2	6.4
Zambia	2013–14	3686	1742	29.5	58.1	13.5	46.4	3.3
Zimbabwe	2015	2156	737	22.5	69.5	16.3	50.7	9.6

NB

^a^prevalence among all adolescents

^b^prevalence among adolescents who had ever had sex.

### Definition of variables

#### Outcome variable

The outcome variable for this study was ‘first adolescent pregnancy’. We defined this as females aged 15 to 19 years who had ever given birth; were pregnant at the time of the survey; or who had ever had a pregnancy terminated. The rationale for looking at ‘first adolescent pregnancy’ was to provide a holistic measurement of adolescent pregnancy, which has been employed in previous studies among adolescents in SSA [22, 23] and globally, where birth and abortion rates (even in countries where data are limited) were each considered important ‘pregnancy outcomes’ [5]. Similar concept was used by Neal, Channon [29] in their study on trends in adolescent first births in SSA, where the authors defined ‘adolescent first births’ as births that occurred before the age of 20 years among women aged 20–24. The need to include pregnancy and abortion data and not just birth rate in the current study has been argued in the transition from the Millennium Development Goals to the Sustainable Development Goals, notwithstanding that underreporting of abortion is inevitable [32]. A sole focus on adolescents who were pregnant at the time of the survey would lead to under-reporting of the actual prevalence of adolescent pregnancy since some girls would have been pregnant previously and have already given birth, and others would have been pregnant and had their pregnancies terminated.

#### Independent variables

We used eleven independent variables: eight were individual level and three contextual level variables. The individual level variables were: age of respondents, marital status, highest educational level, occupation, exposure to media, age at first sex, knowledge of contraceptives and unmet need for contraception. Exposure to media was derived from the proportion of adolescents who either read a newspaper, listened to the radio or watched television at least once per week. The contextual level variables included wealth quintile, place of residence and sub-regions. It should be noted that apart from age at first sex, all the independent variables were measured at the survey date while first pregnancy might have happened years ago. This can lead to the possibility of reverse causality. Detailed description and coding of the variables is available in S1 Table.

### Statistical analysis

We used Stata version 13 to analyse the data. First, we calculated the prevalence of first adolescent pregnancy among all adolescents in the 32 SSA countries using frequencies and percentages. Next, we calculated the prevalence of first adolescent pregnancy among the subset of adolescents who had ever had sexual intercourse. We then conducted bivariate analysis using the chi-square test to assess relationships between potentially explanatory variables and the outcome variable of first adolescent pregnancy. Finally, a two-level multilevel logistic regression model was used to investigate the association between potential explanatory variables and the outcome variable among adolescents who had ever had sex.

The two-level multilevel logistic regression modelling in this study implies that adolescent girls were nested within clusters. Clusters were considered as random effects to cater for the unexplained variability at the individual and household levels [33, 34]. Four models were fitted. Model 0 showed the variance in first adolescent pregnancy attributed to the distribution of the primary sampling units in the absence of the explanatory variables. Model I had the individual level variables while Model 2 contained the contextual level variables. The final model (Model 3) was the complete model that had both the individual and contextual level variables. The Stata command ‘melogit’ was used in fitting these models. Model comparison was done using the log-likelihood ratio and Akaike’s Information Criterion (AIC) tests. The highest log-likelihood and the lowest AIC were used to determine the best fit model (see Table 3). Odds ratios and associated 95% confidence intervals (CIs) were presented for all the models apart from model 0. To ensure there was no strong correlation between the potential explanatory variables, a test for multicollinearity was done using the variance inflation factor and the results showed no evidence of collinearity among the explanatory variables (Mean = 1.24, Maximum VIF = 1.54 and Minimum VIF = 1.06). Categories of the explanatory variables with the lowest prevalence of first adolescent pregnancy among adolescents who had ever had sex were used as reference values in the multivariable multilevel logistic regression analysis.

In terms of applying sample weights, since this was a pooled data analysis, the standard weight variable for the individual recode file (v005) was first de-normalized as follows: v005 × (total female population 15–49 in the country)/ (total number of women 15–49 interviewed in the survey) and then re-normalized so that in the pooled sample the average is 1. This was important because according to the DHS sampling and household listing manual, the normalized weight is not valid for pooled data, even for data pooled for women and men in the same survey, because the normalization factor is country and sex specific [35].

### Ethical approval

Ethical approval was given by individual national institutional review boards and by the Inner City Fund (ICF) International Institutional Review Board. Permission to use the data set was sought from MEASURE DHS. The dataset is available to the public at https://dhsprogram.com/data/available-datasets.cfm. The University of Technology Sydney Human Research Ethics Committee reviewed and approved the conduct of the study (ETH19-3919).

## Results

The prevalence of first pregnancy among all adolescent girls in SSA ranged from 7.2% in Rwanda to 44.3% in Congo. However, among adolescents who had ever had sex, the prevalence ranged from 36.5% in Rwanda to 75.6% in Chad. Table 1 presents the prevalence of first adolescent pregnancy among all adolescent females (15–19 years) as well as for those who had ever had sex in SSA.

### Relationship between individual and contextual level variables and first pregnancy among adolescents who had ever had sex

We examined the correlates of first adolescent pregnancy for the sample of adolescents who had ever had sex (Table 2). Adolescent pregnancy was more likely with increasing age, rural residence, working, being or ever have been married or cohabiting, lower levels of education and non-exposure to media (television, newspaper and radio). Having first sex before 16 years of age, having no knowledge of contraceptives, having no unmet need for contraception, decreasing wealth, and the Central African sub-region were all associated with higher levels of adolescent pregnancy.

**Table 2:** Relationships between individual and contextual variables, and first pregnancy in adolescents who had ever had sex (DHS, 2010–2018).

Variables	Adolescent pregnancy		Chi-square, p-value	Number
	n	%		
**Individual level variables**				
***Age (years)***			(10.6, <0.001)	
15	863	26.0		3320
16	2166	39.2		5521
17	3857	48.8		7898
18	7265	59.9		12134
19	7770	68.2		11400
***Occupation***			(392.9, <0.001)	
Not working	10577	49.5		21375
Working	11345	60.0		18897
***Marital Status***			(28.6, <0.001)	
Never married	6315	30.3		20811
Married/cohabiting/previously married	15608	80.2		19461
***Educational Level***			(8.4, <0.001)	
No Education	6040	68.6		8810
Primary	8733	61.1		14286
Secondary/Higher	7148	41.6		17176
***Exposure to Media***			(671.4, <0.001)	
No	7621	64.9		11750
Yes	14302	50.1		28522
***Age at First Sex***			(537.2, <0.001)	
Less than 16 years	13127	59.3		21980
16–19 years	8795	48.1		18292
***Knowledge of Contraceptives***			(60.8, <0.001)	
Knows no methods	1720	60.7		2836
Knows traditional/modern methods	20202	54.0		37436
***Unmet need for Contraception***			(988.2, <0.001)	
No	6650	68.4		9718
Yes	15273	50.0		30554
**Contextual level variables**				
***Wealth Quintile***			(7.1, <0.001)	
Poorest	5087	66.3		7676
Poorer	5054	62.2		8124
Middle	4719	56.2		8399
Richer	4150	50.1		8288
Richest	2912	37.4		7785
***Residence***			(737.0, <0.001)	
Urban	6875	45.1		15228
Rural	15048	60.1		25044
***Sub-regions***			(337.8, <0.001)	
Western Africa	7806	53.7		14547
Eastern Africa	8081	54.8		14760
Central Africa	5161	58.4		8833
Southern Africa	874	41.0		2131

### Factors associated with first pregnancy in adolescents who had ever had sex in sub-Saharan Africa

In terms of the individual level predictors, the odds of having first adolescent pregnancy in SSA increased with age, with those aged 19 years having approximately 13 times higher odds of experiencing first pregnancy compared to those aged 15 (AOR = 12.81, 95% CI = 11.48–14.29). Adolescents who were working had 9% increase in odds of having first pregnancy compared to those who were not working (AOR = 1.09, 95% CI = 1.04–1.15). Married/cohabiting/previously married adolescents were eight times more likely to have first pregnancy compared to never married adolescents (AOR = 8.30, 95% CI = 7.84–8.78). We also found a 38% increase in odds of having first pregnancy among adolescents with primary education only (AOR = 1.38, 95% CI = 1.30–1.46), compared to those with secondary/higher education. Adolescents who had no exposure to media (television, newspaper or radio) had 8% greater chance of having first pregnancy (AOR = 1.08, 95% CI = 1.02–1.15) compared to those who had media exposure. The odds of having first pregnancy tripled among adolescent girls who had first sex before age 16 (AOR = 3.19, 95% CI = 2.98–3.28) and those who had no unmet need for contraception (AOR = 2.86, 95% CI = 2.69–3.03) but decreased by 30% among those who had knowledge on either modern or traditional contraceptives.

With the contextual level factors, the odds of having first pregnancy doubled amongst adolescents of the poorest wealth quintile (AOR = 2.04, 95% CI = 1.86–2.24), compared to those of the richest wealth quintile. On the other hand, a 12% decrease in odds of having first pregnancy was found among adolescent girls who lived in rural areas (AOR = 0.88, 95% CI = 0.83–0.94) and 36% decrease in odds among those who lived in the West African sub-region (AOR = 0.64, 95% CI = 0.57–0.72), compared to those who lived in urban areas and in Southern Africa, respectively.

With the random effects results, the complete model (Model III), which included all the individual and contextual level factors in the model and had an AIC of 39677.8 and a log-likelihood ratio of -19816.9, was considered as the best fit model for predicting the occurrence of first adolescent pregnancy. The factors associated with first adolescent pregnancy in Sub-Saharan Africa are presented in Table 3.

**Table 3:** Factors associated with first pregnancy in adolescents who had ever had sex in sub-Saharan Africa (DHS, 2010–2018).

Characteristic	Model 0	Model I AOR [95%CI]; p-values	Model II AOR [95%CI]; p-values	Model III AOR [95%CI]
**Fixed effects**				
***Age***				
15		Ref		Ref
16		2.17[1.95–2.42]; <0.001		2.18[1.95–2.42]; <0.001
17		4.59[4.14–5.09]; <0.001		4.66[4.19–5.17]; <0.001
18		7.75[6.99–8.59]; <0.001		7.80[7.20–8.88]; <0.001
19		12.50[11.22–13.93]; <0.001		12.81[11.48–14.29]; <0.001
***Occupation***				
Not working		Ref		Ref
Working		1.07[1.02–1.12]; 0.009		1.09[1.04–1.15]; 0.001
***Marital Status***				
Never married		Ref		Ref
Married/cohabiting/previously married		8.11[7.68–8.57]; <0.001		8.30[7.84–8.78]; <0.001
***Educational level***				
No Education		1.07[0.99–1.56]; 0.091		1.18[1.09–1.28]; <0.001
Primary		1.47[1.39–1.56]; <0.001		1.38[1.30–1.46]; <0.001
Secondary/Higher		Ref		Ref
***Exposure to media***				
No		1.27[1.20–1.35]; <0.001		1.08[1.02–1.15]; 0.008
Yes		Ref		Ref
***Age at first sex***				
Less than 16 years		3.19[3.02–3.37]; <0.001		3.09[2.92–3.28]; <0.001
16–19 years		Ref		Ref
***Knowledge of Contraceptives***				
Knows no method		0.80[0.72–0.87]; <0.001		0.69[0.63–0.76]; <0.001
Knows either traditional/modern		Ref		Ref
***Unmet need for Contraception***				
No		2.92[2.76–3.10]; <0.001		2.86[2.69–3.03]; <0.001
Yes		Ref		Ref
***Wealth quintile***				
Poorest			2.70[2.50–2.91]; <0.001	2.04[1.86–2.24]; <0.001
Poorer			2.34[2.17–2.52]; <0.001	1.99[1.81–2.18]; <0.001
Middle			1.93[1.80–2.08]; <0.001	1.73[1.59–1.89]; <0.001
Richer			1.57[1.47–1.68]; <0.001	1.47[1.36–1.60]; <0.001
Richest			Ref	Ref
***Place of residence***				
Urban			Ref	Ref
Rural			1.22[1.16–1.28]; <0.001	0.88[0.83–0.94]; <0.001
***Sub-regions***				
Western Africa			1.65[1.51–1.81]; <0.001	0.64[0.57–0.72]; <0.001
Eastern Africa			1.78[1.62–1.95]; <0.001	0.85[0.76–0.95]; 0.005
Central Africa			2.32[2.10–2.55]; <0.001	1.16[1.03–1.31]; 0.012
Southern Africa			Ref	Ref
**Random effects**				
Variance (SE)	0.02 (0.01–0.03)	0.01 (0.003–0.032)	0.01 (0.007–0.026)	0.01 (0.004–0.033)
ICC	0.01	0.003	0.004	0.006
Log-likelihood	-27681.3	-20127.6	-26767	-19816.9
LR Test	*χ*^*2*^ = 28.73, p< 0.001	*χ*^*2*^ = 3.54, p = 0.03	*χ*^*2*^ = 11.56, p<0.001	*χ*^*2*^ = 4.25, p = 0.02
AIC	55366.6	40283.2	53554.0	39677.8
N	40272	40272	40272	40272

Exponentiated coefficients; 95% confidence intervals in brackets; AOR adjusted Odds Ratios CI Confidence Interval.

N = Sample size; SE = Standard Error; ICC = Intra-Class Correlation; LR Test = Likelihood ratio Test; AIC = Akaike’s Information Criterion.

Model 0: Null model without any explanatory variable.

Model I: Adjusted for the individual level variables.

Model II: Adjusted for the contextual level variables.

Model III: Adjusted for individual and contextual level variables.

## Discussion

To our knowledge, this is the first study that has sought to examine the prevalence of first adolescent pregnancy and its associated factors across 32 sub-Saharan African countries. We found that the prevalence of first adolescent pregnancy was highest in Congo and lowest in Rwanda. Among adolescents who had ever had sex, we found that increasing age, working, being married/cohabiting, having primary education only, early sexual initiation, knowledge of contraceptives, no unmet need for contraception and poorest wealth quintile were associated with having first adolescent pregnancy. By contrast, adolescents who lived in rural areas and in the West African sub-region had lower odds of having first pregnancy.

The high prevalence of first adolescent pregnancy in Congo and in Central Africa confirm the findings of reports by UNICEF [36] and UNFPA [7]. One possible reason for this is that Congo has one of highest rates of child marriage globally, with one in three girls married before their 18^th^ birthday and 7% married before the age of 15 [37]. Several other studies have found an association between child marriage and adolescent pregnancy [38–40]. Most girls who experience child marriage have no education, live in poor households and often in rural areas, increasing their odds of engaging in behaviours that put them at risk of pregnancy [41].

Being married or in relationship was also identified as a factor associated with first pregnancy among adolescent girls who had ever had sex in SSA. This is supported by previous studies [26, 42]. One of the plausible reasons for this is that marriage/cohabitation predispose adolescent girls to pregnancy since they increase their desire to have children. This becomes even stronger in most sub-Saharan African countries, where adolescent girls may face social pressure to marry and, once married, to have children. On the other hand, other studies have shown that some adolescent girls are given into marriage or end up cohabiting after pregnancy [43, 44].

In terms of the relationship between place of residence and first adolescent pregnancy, the odds of having first pregnancy was high among adolescents who lived in rural areas in the Model that had only the contextual level factors (Model II). However, in the model that adjusted for both the individual and contextual level factors, a reverse association occurred. This could mean that individual level factors play a role in the association between place of residence and first adolescent pregnancy.

Adolescent girls with knowledge of contraceptives were more likely to have first pregnancy. Although apparently counter-intuitive, it is possible that knowledge of contraceptives occurred after a pregnancy had occurred. Other explanations include that reported knowledge was superficial and that adequate knowledge about the range and use of contraceptive methods was lacking [45]. Alternatively, pregnancy might have occurred in spite of contraceptive knowledge due to the desire or social pressure to become pregnant and was not mitigated by outside incentives to delay childbearing [46]. Societal norms such as condemning early engagement in sex, pregnancy and use of contraceptives among unmarried adolescents can also present major obstacles to contraceptive use [47]. Moreover, information on contraceptives may be incorrect and filled with misconceptions, especially when stemming from unreliable rather than trust-worthy sources of information [12, 48, 49]. Studies from SSA have shown that higher knowledge of contraceptives, especially among adolescents, does not always lead to higher utilization of contraceptives [48, 50, 51] and that most adolescents with high knowledge of contraceptives often face barriers in accessing and using contraceptives, including stigma and discrimination by healthcare providers and fear of side effects [48, 52, 53]. Other possible reasons for the finding is that knowledge of contraceptives can occur after childbirth/abortion [12, 54, 55].

Having no unmet needs for contraception was also shown to be associated with first adolescent pregnancy in our study. The possible reason for the seemingly counter-intuitive finding could be that adolescent girls may have different fertility intentions after pregnancy, abortion or childbirth [56]. Other possible explanations for this include that adolescent girls may have used traditional or folkloric methods rather than modern contraceptives. Contraceptive failure, incorrect and inconsistent condom use as well as non-use of contraceptives can lead to unplanned pregnancy [57].

Higher levels of education were linked with lower likelihood of having first adolescent pregnancy in SSA, a finding consistent with much of the existing literature [25, 58, 59]. With greater education, adolescents’ opportunities to avoid early childbearing may improve due to increased knowledge and agency in prevention of unintended pregnancies [25]. Adolescents with higher levels of education are also more likely to delay onset of sexual relations and marriage; are more empowered and better informed about those fundamental and legal rights that are indispensable in decision-making about healthy living including optimal timing of marriage and pregnancy [58]. Another reason for this finding could be the possibility of reverse causality as adolescents with children might have to drop out from school.

Adolescent girls who were working were more likely to experience first pregnancy compared to those who were not working. Several other studies have also found the risk of adolescent pregnancy to be higher among adolescent girls in employment [24, 60], perhaps because female adolescents who are not working may be in school. Most of these students may have access to sexuality education, which has been found to reduce the likelihood of adolescent pregnancy [61–63]. The likelihood of repeated pregnancies among out-of-school adolescents is very high with high prevalence of risky sexual behaviour reported among out-of-school adolescents [64, 65]. The possibility of reverse causality may also account for the high prevalence of first pregnancy among working adolescents as getting pregnant/having a child might influence the probability of working [66].

Adolescent girls in SSA who were exposed to media (television, newspaper or radio) had lower odds of having first adolescent pregnancy. This supports the findings of previous studies [19, 25, 67, 68]. Adolescent girls who are exposed to media may have greater access to SRH information [69, 70]. Such information can empower them in relation to their sexual rights and choices. Sexual and reproductive health communications through the media may promote healthy sexual development and reduce sexual risk-taking behaviours [71]. On the other hand, studies have also found that exposure to media can be linked to adolescents engaging in behaviours that put them at risk for adolescent pregnancy [72, 73].

Finally, later sexual debut was linked to lower rates of having first adolescent pregnancy in SSA, as in other studies [42, 59, 74]. The possible reason for this finding is that later sexual debut is associated with less time of exposure to pregnancy [75]. Other reasons could be that contraceptives are more often used effectively to prevent pregnancy among adolescent girls who engage in later sexual debut, and older adolescent girls might be more able to negotiate safer sex with their partners [59].

### Limitations of the study

Caution is required in interpreting this study’s findings because the study’s cross-sectional design did not permit the examination of causal relationships between these variables and rates of adolescent pregnancy in SSA. The use of composite data to examine the influences on adolescent pregnancy in 32 SSA countries is a further limitation, taking into consideration the heterogeneity of these countries and their cultures. However, this was addressed to some extent by controlling for the effect of the sub-regional variable in the multilevel logistic regression analysis. The pooled data included surveys spanning close to a decade and experiences may vary across a decade. Moreover, including adolescents who had ever had a pregnancy terminated as part of the measure of adolescent pregnancy is likely to lead to bias in the findings since it has been found that data on pregnancy termination in the DHS are often of poor quality and under-reported [76]. Again, for some participants, questions asked were in reference to issues that occurred after pregnancy, while for others, the questions asked were in reference to current pregnancy. For this latter group, current pregnancy may have affected their reported knowledge and behaviour. Finally, apart from age at first sex, data on the explanatory variables included in this study refer to the time of the surveys, and may differ to the experience at the time of pregnancy. This can lead to reverse causation, where, for example, education may have been discontinued, marriage occurred or knowledge of contraception been acquired after pregnancy.

### Policy and public health implications

Our findings have implications for policy, public health and further research. The prevalence of first adolescent pregnancy in SSA varies widely, with high prevalence among adolescents in Central Africa. Understanding the individual and contextual level factors associated with first adolescent pregnancy, while controlling for individual countries, adds to existing literature and can help support improvement in social policy development. The success of policies would depend on cultural and social change, coupled with engagement of adolescents and stakeholders in adolescent sexual and reproductive health. There is evidence that policies exist across much of SSA that support comprehensive sexuality education and sexual and reproductive health services accessibility in most countries in SSA. However youth involvement in policy formulation, and plans for implementation, monitoring and evaluation are inadequate [77]. Such policies should also aim at eradicating child marriage, which puts adolescent girls at risk of pregnancy [78]. In the long term, understanding the complexities that exist beneath predictors of adolescent pregnancy and improving the implementation of policies will help to achieve Sustainable Development Goal 3 that seeks to ensure healthy lives and promote well-being for all at all ages. Our findings provide a basis for future research on adolescent pregnancy in the region. Future studies should examine the predictors of adolescent pregnancy using prospective study designs which can address some of the major limitations of the current study. Additionally, the use of qualitative research can provide rich data to explain the complexities of adolescent pregnancy in differing cultures of SSA.

## Conclusion

Concerns remain about the high level of first adolescent pregnancy across SSA. Building on previous research into factors associated with adolescent pregnancy in SSA, we found that age, occupation, marital status, level of education, early sexual initiation, knowledge of contraceptives, unmet need for contraception and wealth quintile are associated with first adolescent pregnancy in SSA. To ensure that SDG 3 can be realised by 2030, there needs to be investment in policy implementation and evaluation and engagement with stakeholders of adolescent sexual and reproductive health.

## Supporting information

S1 Table Description of the study variables. (DOCX)
