# Supplementary material for: Prevalence of first adolescent pregnancy and its associated factors in sub-Saharan Africa: A multi-country analysis
Source: PLoS One. 2021 Feb 4;16(2):e0246308. doi: 10.1371/journal.pone.0246308 (PMC7861528; doi:10.1371/journal.pone.0246308)
Supplement: S1 Table — (DOCX) [file pone.0246308.s001.docx]

**S1 Table: Description of the Study Variables**

| **Variables** | **Question/Description** | **Response options and recoding** |
| --- | --- | --- |
| **Individual level variables** |  |  |
| ***Age*** | How old were you at your last birthday | 1 = 15  2 = 16  3 = 17  4 =18  5 = 19 |
| ***Occupation*** | Standardized respondent's occupation groups. | 0 = Not working  1 = Working |
| ***Marital Status*** | 1. Are you currently married or living with a man as if married 2. Have you ever been married or lived together with a man as if married | 0 = Never Married  1 = Married  2 = Cohabiting  3 = Previously married |
| ***Educational Level*** | What is the highest level of school you attended: primary, middle, JSS/JHS, Secondary, SSS/SHS, or higher? | 0 = No education  1 = Primary  2 = Secondary/Higher |
| ***Access to Media:*** Derived from three questions related to exposure to TV, radio and newspaper | 1. Do you read a newspaper or magazine at least once a week, less than once a week or not at all? 2. Do you listen to the radio at least once a week, less than once a week or not at all? 3. Do you watch television at least once a week, less than once a week or not at all? | 0 = No (not at all for all three)  1 = Yes (at least once a week+ less than once a week for either TV, radio or newspaper) |
| **Sexual and Reproductive Health Variables** |  |  |
| ***Age at First Sex*** | How old were you when you had sexual intercourse for the very first time? | 1 = Less than 16 years  2 = 16-19 years |
| ***Knowledge of Contraceptives:*** Knowledge of any method is classified into modern, traditional and folkloric methods. Modern methods are Pill, IUD, Injections, Diaphragm, Condom, Female  Sterilization, Male Sterilization, Implants, female condom, Foam/Jelly and lactational  amenorrhea. Traditional methods are Periodic Abstinence (Rhythm), Withdrawal, and  Abstinence. Folkloric methods are the category "other". | Now I would like to talk about family planning-the ways or methods that a couple can use to delay or avoid a pregnancy. Have you ever heard of (METHOD)? | 0 = Knows no method  1= Knows traditional/folkloric method  2 =Knows modern method |
| ***Unmet need for Contraception*** | Unmet need for contraception categorizes women according to whether they have an  unmet need or a met need, to space or to limit their future births | 0 = No  1 = Yes |
| ***Contextual level variables*** |  |  |
| ***Place of Residence*** | Type of place of residence where the respondent was  interviewed as either urban or rural. | 1 = Urban  2 = Rural |
| ***Wealth Quintile*** | The wealth index is a composite measure of a household's cumulative living standard. | 1 = Poorest  2 = Poorer  3 = Middle  4 = Richer  5 = Richest |
| ***Sub-regions*** | Burkina Faso, Benin, Cote dlvoire, Ghana, Gambia, Guinea, Mali, Nigeria, Niger, Sierra Leone, Senegal, Togo=1  Burundi, Cameroon, Ethiopia, Gabon, Kenya, Comoros, Malawi, Rwanda, Tanzania, Uganda, Zambia, Zimbabwe=2  Angola, Congo DR, Congo, Liberia, Chad=3  Lesotho, Namibia, South Africa=4 | 1=West Africa  2=East Africa  3=Central Africa  4=Southern Africa |
